# Is Personality Profile a Relevant Determinant of Fatigue in Multiple Sclerosis?

**DOI:** 10.3389/fneur.2015.00002

**Published:** 2015-02-04

**Authors:** Herbert Schreiber, Michael Lang, Kristina Kiltz, Charlotte Lang

**Affiliations:** ^1^Neuropoint Patient Academy, Neurological Practice Center, Ulm, Germany; ^2^Neuro Trans Data (NTD) Study Group on Multiple Sclerosis, Neuburg, Germany; ^3^Department of Neurology, Marienhospital Stuttgart, Stuttgart, Germany; ^4^Department of Neurology, Marienhospital Düsseldorf, Düsseldorf, Germany

**Keywords:** multiple sclerosis, fatigue, personality assessment, depression, coping behavior, anxiety

## Abstract

The origin and pathophysiological background of multiple sclerosis (MS)-associated fatigue is poorly understood. There is no unifying concept of its nature and its determinants to date. This paper reviews possible influences of factors determining personality profile on fatigue in MS. Likewise, the role of psychological factors and their interaction with personality to promote fatigue is discussed. Current data suggest that fatigue, especially in early MS states, may be influenced by vulnerable personality traits and personality-associated features. Among them are depressive disease coping, avoidance behavior and inhibition, irritability, less extraversion, neuroticism, lower reward responsiveness, and somatization behavior. However, among the validated personality factors, no genuine influences that are independent of depression have been documented. From a psychological perspective, depressiveness, anxiety, and somatization may be relevant mediators of fatigue. Interesting to note that in early MS, a psychiatric diagnosis is significantly more likely than on a later stage of the disease and that fatigue and motivation might share neural circuits. It is hypothesized that psychological factors promote fatigue in MS by psychological distress and sustained neuroendocrine and neurovegetative stress response. Despite the limitations of data discussed in the paper, personality research might help to disentangle specific promoting factors of fatigue in MS. Further research efforts are warranted since they might open ways to early psychological intervention of MS-associated fatigue. This is all the more important since medication is insufficient until now.

## Introduction

Fatigue is one of the most prevalent symptoms of multiple sclerosis (MS). It may already be present at early stages and is often the cause for psychosocial and occupational problems. It even happens that otherwise unaffected MS patients cannot work anymore because of fatigue. Fatigue can be defined as either a feeling, i.e., a subjective lack of physical and/or mental energy that is experienced by the individual or caregiver to interfere with usual or desired activities (1), or as a performance decrement, i.e., an inability to complete mental or physical tasks at normal performance level (2). Mental fatigue, in this context, is a transient decrease in optimal cognitive performance resulting from prolonged periods of cognitive activity and manifesting as concentration deficit and cognitive slowing. Motor fatigue, or muscle fatigue, is the inability of a muscle to perform continuously in the sense of a “use-dependent conduction block” (3). The onset of muscle fatigue during physical activity is mostly gradual, and it can be reversed by rest. The same is true for mental fatigue. With respect to basic mechanisms, fatigue can be attributed to the temporary loss of power to respond in sensory receptors, motor end organs, or complex behavioral networks induced by continued stimulation.

Fatigue may have physical, mental, and probably psychological causes. However, insight in its pathophysiology is very limited to date, and there is no unifying concept of its nature and its determinants until today. Even its definition remains controversial among clinicians and researchers since most questionnaires and studies rely on subjective evaluation, i.e., patient self-report. Thus, progress continues to be hampered by unsolved questions related to terminology and assessment (4).

When looking at the multitude of influences that have been claimed to cause fatigue, MS-associated fatigue is less likely a unitary symptom than a construct integrating multiple facets that might emanate from different mechanisms of origin (2, 5). Pro-inflammatory cytokines, autonomic and neuroendocrine dysfunction, a.o. blunted hypothalamic–pituitary–adrenal axis (HPA) axis, nerve conduction block, and inadequate cortical and subcortical activation patterns have been implicated as the pathophysiological key factors. But it seems true that a highly complex interplay of pathophysical, behavioral, and psychological factors contributes to the appearance of fatigue in MS. The most influential candidates in the psychological domain are depression, anxiety, and disease coping. Moreover, there is growing evidence that personality traits interact as behavioral determinants. Personality research in fatigue has been triggered by early studies suggesting a link between premorbid personality characteristics and fatigue in healthy individuals (6, 7).

Despite different concepts, personality is most commonly defined as “that pattern of characteristic thoughts, feelings, and behaviors that distinguishes one person from another and that persists over time and situations” (8, 9). Thereby, the term “personality trait” is thought to refer to enduring personal characteristics that are revealed in a particular pattern of thoughts, feelings, and behavioral modes in a variety of situations. Since personality traits are relatively stable over time, rather specific among individuals and influential predictors of behavior, they are also used to get insight in the patients’ individual response to challenging disease experiences.

## Relationships between Validated Personality Traits and MS Fatigue

There are only a few studies addressing the relationship between MS-associated fatigue (MSF) and personality traits so far. Merkelbach et al. (10) found a higher prevalence of altered personality factors, i.e., higher neuroticism scores and reduced extraversion in relapsing-remitting multiple sclerosis (RRMS) versus healthy controls (HC). MS patients with fatigue (MS-F) presented more emotionally instable, hypersensitive, and introverted compared to those with lower fatigue scores. The authors concluded that personality factors contribute to fatigue in MS and even may exert more influence than physical deficits. Penner et al. (11) also found higher scores of neuroticism and reduced extraversion related to fatigue in 41 MS patients and 41 controls. Likewise, depression turned out to be a main influencing factor of fatigue, the association between mental fatigue and depression being particularly strong. When including depression as a covariate into the regression analysis, the significant influence of personality traits on fatigue was no longer present. On the other hand, a decreased level of action control, i.e., the ability of maintaining own aims and goals against competing external stimuli, persisted as significant influential factor on fatigue. The authors concluded that in MS patients with fatigue, cognitive and motivational control of behavior might work less efficiently and be controlled to a higher degree by situational triggers, as is the case in state orientation behavior. Since no relation was found between fatigue and action control within the control group, the authors speculated that disturbances of action control might be specific for MS-related fatigue (MSF). Kiltz et al. (12) by evaluating physical, cognitive, and psychological dimensions of fatigue in 102 early MS patients, among them 48 MS-F, 54 MS-NF, and 29 HC, revealed highly significant differences between fatigued and non-fatigued MS patients in various aspects of personality and disease coping. The respective personality traits correlating with fatigue were: less performance orientation, minor self-content, more inhibition, irritability and aggressiveness, more demand and physical complaints, less extraversion, and more neuroticism. The respective disease coping factors were significantly higher depressive coping and more extenuation/wishful thinking. The authors concluded that premorbid, not intrinsically MS-related factors (personality, disease coping) might be essential contributors to fatigue, especially in the early phase of MS. Since fatigue also scored higher with more severe disease, future task would be to disentangle the contributions of central nervous system (CNS) deficit and psychological factors, especially personality, disease coping, depression, and anxiety, to the expression of fatigue in MS. In a subsequent longitudinal study, when monitoring the identical parameters in the same group of patients and re-evaluating them by multivariate analysis after 2 years (13, 14), it was found that none of cognitive parameters was differentially expressed between groups. But it were the same personality traits, disease coping factors, and depression that again discriminated between MS-F and MS-NF patients. Moreover, the personality profile remained unchanged over time despite the experience of a chronic disease. Analysis with mixed linear models provided evidence that fatigue was not only correlated to but also directly influenced by several personality traits (i.e., performance orientation, demand, extraversion), depression, disease coping, and disease status as assessed by Expanded Disability Status Scale (EDSS), but not by disease duration. The fact that most fatigued patients expressed both dimensions of fatigue (physical and cognitive) prompted the authors to conclude that both fatigue dimensions are rather complementary than independent entities.

In sum, the currently available studies suggest that fatigue, especially in early MS, may be influenced by vulnerable personality traits. Since personality traits are commonly seen as enduring determinants of behavior, it is probable that these characteristics in personality profile are not intrinsically linked to MS. This would mean that they should be able to cause comparable reactions in other chronic diseases. Alternatively, they might also be a consequence of disease coping. But due to a lack of valid premorbid data, this question cannot be settled to date.

However, current data cannot prove a genuine influence of personality factors on fatigue that is independent of depression. Personality seems to interfere with or work through psychological factors (depression, anxiety) that generate fatigue. This is outlined in more detail later. A limitation of all current data is the fact that they are based on subjects’ subjective experience indexing trait fatigue over longer time periods as assessed by self-report questionnaires. No objective performance measurement of fatigue and no validation of the characteristic MS-related performance decrement have been done in correlation to personality traits so far. Therefore, present data on the personality profile in MS fatigue may primarily index psychological facets of fatigue, i.e., its “trait” character, while fatigue caused by functional brain alterations may represent more “state” forms of fatigue.

## Relationship between Personality-Associated Features and Fatigue in MS

The assessment of personality-associated features in MS patients is relevant because they may represent part of the “intermediate phenotypes” of fatigue in MS. This concept is a modification of that of “endophenotypes,” which comes from genetic epidemiology and is mainly used in psychiatric genetics to converge behavioral symptoms to phenotypes with straight genetic background. Both concepts are closely related to one another representing approaches to find basic genetic–pathophysiological factors and psychological–behavioral drives of complex syndromes.

The intermediate phenotype (endophenotype) construct is therefore an appropriate approach in the field of behavioral neurology to index those basic neuropsychological and behavioral processes that might play a role in the development of the complex syndrome “fatigue.” Moreover, such an approach might shed light on (mal-)adaptive coping behavior and therefore contribute to explain fatigue states with respect to personality influences. The literature contains a few studies related to MS. Van der Werf et al. (15) explored the role of helplessness as a mediator between neurological disability and psychobehavioral factors. They found that more neurological impairment and more emotional instability created more helplessness, the latter being associated with more experienced fatigue and depressive mood. In support of this, emotional instability that characterizes the personality trait neuroticism has repeatedly been related to fatigue. Hyphantis et al. (16) claim that specific personality features, especially defense style and ego strength, may be considered as indicators of premature exhaustion of patients’ vital energy. Interestingly, they found that the odds of being assessed with a psychiatric diagnosis were 9.3 times higher among patients with recent-onset MS compared to those with long-term disease. This highlights the problem of disease coping after revealing the diagnosis of MS to patients. Jopson and Moss-Morris (17) and Skerret and Moss-Morris (18) suggest that the work-up of a strong “disease identity,” i.e., of a high internal representation, is an important predictor of physical and cognitive fatigue. They argue that the more MS patients tend to subjectively attribute every deficit and misfeeling to the MS, the more they are fatigued. One can speculate that such a behavior is dependent on personality features, but a direct correlation to objectively assessed personality traits has not been investigated to date. Also the role of spiritual beliefs, control beliefs, and personality in MS fatigue has been studied. Thus, Wahlig (19) found in a doctoral dissertation that fatigue in MS patients was inversely correlated with “I feel peaceful” during an observation period of 3.5 years. However, the relationship between spiritual beliefs and personality has not been specified. Recent work of Pardini et al. (20) has focused on the motivational system of MS patients evaluating behavioral activation and inhibition on the basis of Gray’s theory of personality (21). It conceptualizes personality as being represented by two basic dimensions of activity control, i.e., a behavioral activation system (BAS) and a behavioral inhibition system (BIS). Greater BAS sensitivity is thought to foster engagement in goal-directed efforts, while BIS is thought to be aversive causing negative experiences during goal-directed activity. Thus, when assessing fatigued MS patients’ reward perception as important part of the BAS, Pardini’s group showed lower reward responsiveness to be present in fatigued MS compared to fatigue-free patients. And lower reward responsiveness scores were found to be associated with Modified Fatigue Impact Scale (MFIS) scores at baseline and to correlate with minor fatigue reduction after treatment. The authors conclude that disturbed reward-related cognition may be one of the “key cognitive underpinnings” of MSF. These findings add to the literature of possible relationships between different personality features and fatigue in MS. Other studies also provide evidence for an overlap between motivational system and fatigue level. Thus, in fatigued subjects, an increased reward for the completion of a task has been shown to reduce some of the effects of fatigue in behavioral performance and neurophysiological testing of centralized fatigue (22, 23). In this context, it is interesting to note that fatigue and the motivational system might share some common neural circuits. For instance, lesions in the ventromedial prefrontal cortex could be related to increased fatigue perception levels and also to deficits in the evaluation of outcomes as rewarding or non-rewarding (24). Most interestingly, depression has been excluded as a relevant confounding factor in Pardini’s MS patients who were required to show normal Hospital Anxiety and Depression Scale scores (HADS) to enter the study. Therefore, motivational testing in the context of reward perception seems to represent a distinct entity that is not merely an efflux of depression.

It sum, it can be said that the studies hitherto available on personality features in MS-associated fatigue provide promising first approaches to the field. However, more systematic research is warranted to further substantiate how and to which extent personality-associated intermediate phenotypes might contribute to the generation of fatigue in MS and other diseases. Again, however, it seems to be only the trait variant of fatigue that can be addressed in this context. Methodological limitations remain and imply also the fact that not all personality features investigated to date are sufficiently validated. Nevertheless, the currently available data give a first impression of the possible significance of a covertly vulnerable personality structure that results in the development of MSF by causing “maladaptive” disease coping and psychological distress.

## Interaction of Depression and Personality Traits in MS-Associated Fatigue

A decisive question is whether we are essentially assessing some sort of state depression instead of enduring personality traits when evaluating personality structure in MSF. This is relevant because both disorders share a high prevalence among MS patients, which makes coincidence in individuals probable, and it may be difficult to differentiate between coincidence and interaction. Moreover, no reliable data are available to date with respect to the premorbid personality structure of fatigued MS patients. Accordingly, their personality profile as assessed after the onset of the disease might not be “genuine” but already be altered by adaptive behavior and depression. Supporting evidence for a high interference of certain personality traits with depressiveness can be drawn from observations with patient groups other than MS. In patients with chronic fatigue syndrome (CFS), the subgroup with concomitant depressive disorder accounted for most of the personality pathology (25). And neuroticism was found to account for 22% of the variance of depression in MS patients after 3.5 years follow-up (19). Likewise, Penner et al. (11) defined depression as an influential factor of personality traits. Various personality changes being related to MS fatigue were no longer significant after control of depression as a covariate. Also in the patient sample of our group, MSF was significantly influenced by the factor depression. But when looking at individual expressions of depression in fatigued patients, it turned out that, despite higher mean values, depression in nearly all cases scored below the clinically relevant threshold raising the question of clinical relevance (14).

These observations suggest that fatigue and depression in MS, despite interacting with each other, are essentially distinct entities. The opinion that MSF is a mere expression of a somatic depression with vital deficit is also not compatible with clinical experience. First, MSF is mostly of shorter duration, in contrast to more persistent fatigue associated with depression, and it is closely activity-related, i.e., shows performance decrement. Second, its aggravation by heat is rather unique for MS and not seen in depression. Against this background, it is comprehensible that no consistent beneficial effect of antidepressant drugs in MS has been found so far.

It can be hypothesized from the current data that MS-associated fatigue is not congruent with depression, but that depressive mood may promote fatigue. In line with this hypothesis, MS-related mental fatigue has been shown to be preceded by reduced motivation and emotional distress (26). In the proportion of MS patients that are fatigued, depression and anxiety may be the “interface” between a vulnerable personality structure facilitating maladaptive disease coping behavior, and fatigue. Interpretation of current data, that are not fully consistent, suggests that a personality characterized by emotional lability (neuroticism), inhibition/avoidance, inflexible cognitions, and less open-mindedness (extraversion) is more prone to “maladaptive” disease coping behavior, anxiety, and depression, than a resilient personality. Depression, in turn, may aggravate feelings of fatigue that again are the primary pick-up criterion of conventional fatigue questionnaires. It is interesting to note in this context that anxiety, depression, and fatigue are not only highly prevalent in MS but tend to cluster together. A recent study has emphasized that the prevalence of the three factors is high in MS, with depression rarely occurring alone or without concurrent anxiety and/or fatigue (27). Notably, the psychological dimension of fatigue has especially been advocated for fatigue feelings over extended time (trait fatigue) and in early MS when structural and functional brain deficits are not prevalent. In later disease stages, physical dimensions of fatigue, more linked to performance decrement (4) may gain importance.

## Personality Profile in MS-Related Fatigue and Chronic Fatigue Syndrome

When discussing the personality profile of fatigued MS patients, it is rewarding to look at the CFS. It is a disorder without obvious neural damage and without a consistent biological marker that, at first glance, shares striking similarities with MSF in clinical picture including vital deficit beyond fatigue, sleep disorder, and attention deficit. Unlike many depressed MS patients, fatigued MS patients are usually not dominated by negative affect (28) resembling CFS subjects who tend to make physical attributions for their deficit (29). And the dominant symptom of CFS is pervasive fatigue, but interestingly, less performance decrement is occurring in CFS patients than seen in fatigued MS patients.

The question, whether there are similarities in the personality profile between fatigued MS and CFS patients, has been addressed in a few studies. Early work concentrated on psychiatric aspects. Thus, Pepper et al. (30) found no differences concerning personality disturbances between CFS and MS fatigue patients, but more frequent depression in CFS, especially following the onset of the disease. A study comparing relative rates of personality disturbance in CFS, fatigued MS, and depressed patients revealed higher rates of personality disorders in all three patient groups compared to controls with depressed patients showing the highest scores and MFS and CFS patients medium scores. However, personality alterations in the CFS group did not differ from that exhibited by MS patients (25). Christodoulou et al. (31) evaluated personality profiles on the basis of Cloninger’s four basic dimensions of personality in CFS compared to MS patients and HC. MS patients were unique in terms of having lower Persistence Level than CFS patients and HC, and comparable with CFS patients in terms of increased sensitivity to negative stimuli (i.e., higher levels of Harm Avoidance) and lower levels of Reward Dependence as compared to HC. The reduced Persistence Level in MS patients has been interpreted by the authors according to Cloninger’s theory as the tendency of the individual to persevere in behaviors that have been previously associated with reward or relief from punishment. Taillefer et al. (32) examined personality, depression, and illness worry in CFS versus MS outpatients and detected no differences with respect to neuroticism and depressive symptoms. On the other hand, CFS patients showed a significantly higher illness worry index than MS patients. The latter, however, were not all in a state of fatigue.

Summing up, current data do not substantiate any essential differences concerning personality profiles in CFS and fatigued MS patients. Alternatively, CFS patients could be distinguished from depressed patients on clinical grounds and psychometric testing. In a study comparing CFS and depression, the CFS patients were characterized by lower ratings of their health status, stronger illness identity, making external attributions of their illness, and distortions in thinking that were specific to somatic experiences. They were more likely than depressed patients to cope with their illness by limiting activity levels, and somatic illness identity turned out to be the most significant predictor of ongoing fatigue (33). In view of such findings, it has been suggested that CFS and MSF might share similarities as a somatization disorder. But the body of data on this issue is mostly speculative to date so that valid conclusions cannot be drawn. Otherwise, there is growing evidence to implicate somatic mechanisms (causative or adaptive) in CFS, especially abnormalities of the HPA axis with altered hormonal stress response. This involves reduced adrenocorticoid hormone (ACTH) response, hypocortisolism, and increased serotonin neurotransmission that, very noteworthy, are contrasting with patterns observed in depressed patients (34). It can be concluded from the data that psychological and somatic factors coexist and may interact to produce the complex behavioral correlate of fatigue in CFS.

## How may Personality Traits and Personality-Associated Factors Contribute to MS-Related Fatigue?

One important pathway by which personality factors may provoke fatigue is “maladaptive” disease coping. This may cause psychological distress and, prompt various psychological, neuroendocrine, and neurovegetative dysregulations that ultimately result in fatigue. The term maladaptive is thought to index coping behavior that is not primarily based on problem-solving but on emotional reactions involving negative feelings, anxiety, exaggerations, and negative cognitions. Personality is known to determine to a high degree the choice of coping strategies (35), although the impact of situative factors is acknowledged as well. Thus, coping strategies have been found to differ between disease populations and HC (36). Since MS is a chronic and potentially disabling disease that affects patients primarily in younger age, the confrontation with such a diagnosis has to be considered an extremely stressful event that requires adequate coping. In such situations, personality factors that might provoke inadequate modes of adaptation are detrimental. In this context it is important to highlight early work of Folkman and Lazarus (37) who stressed the significance of emotional coping strategies for challenges that act outside of subjects’ control (severe disease) and the importance of cognitive coping strategies for challenges within subjects’ control (for real problem-solving). In MS disease, emotional coping strategies have been found to prevail in the early stages, while rational (cognitive) strategies gain importance in later stages (38). In support of this, Goretti et al. (39), when exploring coping strategies among MS patients, found that problem-focused strategies are less likely used and avoiding strategies adopted more often.

It can be concluded from this that emotional coping and avoidance behavior entail more risk of psychological distress than cognitive coping strategies, and pave the way to sustained stress responses and ultimately fatigue. This view is supported by recent work of Nielsen-Prohl et al. (40) providing evidence that personality-related volitional coping competences required by daily stressful situations are a relevant factor for depressive mood in individual MS patients. The crucial role of personality traits for the development of psychological problems in MS has also been advocated by other authors (16, 41–43). Especially Rabinowitz and Arnett (43) were able to show in a longitudinal study that depression in MS is dependent on coping styles and that psychological and cognitive status and coping behavior affect each other. Thus, “adaptive” coping protected MS patients from experiencing depression, but when individuals used maladaptive coping, coexisting cognitive dysfunction put them even more at risk for depression. Results suggest that tertiary problems, for example cognitive dysfunction, add to the risk of depression due to an independent negative effect on coping. A personality profile described in the literature as accentuated by inhibition/avoidance, irritability, and aggressiveness, i.e., showing less extraversion and more emotional lability (“neuroticism”), would fit into this model. Such personalities, though not being pathological in terms of a personality disorder, may soon come to a state of psychological distress entailing irritability, depressiveness, and anxiety when facing the diagnosis of MS.

In this context, it may also be asked whether somatization behavior might play a role in fatigue. Despite being speculative, there are several analogies to consider. First, the nosological and etiological boundaries of patients with complaints of chronic fatigue have not been clearly delineated so far. Various disorders are subsumed and patients with chronic fatigue are likely to have comorbid affective, anxiety, and somatoform symptoms (44). Second, somatization patients have a tendency of being hypersensitive to stimuli and more aware of bodily sensations, thinking catastrophically about their physical sensations, and having increased emotional distress, all of which may enhance physical symptoms. Their state of increased reactivity has even been documented neuro-physiologically (45). Third, an association of fatigue and somatization disorder with hypocortisolism has repeatedly been reported (46) with the most consistent correlate being reduced cortisol response from dysregulation of the HPA axis (47). A possible cause of patients’ hypersensitivity may be repeated or prolonged exposure to stress. Therefore, it has been argued that somatization patients overstrain their stress response system for a long time resulting in blunted HPA axis function (48). HPA dysfunction again represents the final pathway that has been implicated as an important pathophysiological cause of fatigue (49).

From a psychological perspective, it can be summarized that depressiveness, anxiety, and somatization may be relevant mediators and interfaces to fatigue in MS. But current research suggests that this psychological interface is less likely to act by means of a full-scale somatic depression than by influencing a more complex network of psychological and somatic factors. These involve maladaptive disease coping, inadequate stress response, altered central immune mechanisms (pro-inflammatory cytokines), and neuroendocrine changes (HPA axis). The latter, in turn, may directly be influenced by demyelinating lesions, axonal damage, and altered immune status (upregulation of pro-inflammatory cytokines). The assumption that pro-inflammatory cytokines may pathogenetically be relevant for fatigue relies on laboratory findings that (1) pro-inflammatory cytokines (IL-1, IL-6, TNF-alpha, and Il-12) were positively correlated with fatigue in MS (50–52), (2) TNF-alpha was correlated with the severity of fatigue in MS (52), and (3) TNF-alpha in animal experiments was able to trigger a fatigue syndrome (53). Again there are cytokine–neuroendocrine interactions by which central immune reactions gain influence on the HPA axis.

Thus, psychological and somatic factors seem to converge to final pathways to create fatigue. MS fatigue according to this concept would integrate complementary somatic and psychological causes and be the end-product of an interplay of multiple factors that, in the individual case, change in loading and composition according to disease stage.

## Conclusion

Current data suggest that fatigue, especially in early MS states, may be influenced by vulnerable personality traits and personality-associated features that are premorbid factors and not intrinsically linked with MS. Among them are depressive disease coping, avoidance behavior, inhibition, irritability, less extraversion, neuroticism, disturbed reward responsiveness, and somatization behavior. However, among the validated personality factors no genuine influences being independent of depression have been found. From a psychological perspective, depressiveness, anxiety, and somatization behavior may therefore be relevant mediators of fatigue promoting it by psychological distress and sustained neuroendocrine and neurovegetative stress response.

Personality research on fatigue in MS is attractive because it might open ways to early psychological intervention targeting unfavorable disease cognitions and coping. This is all the more important since medication is insufficient to date. Further research on the expression and interaction of personality profiles, depressive mood, anxiety states, and disease coping orientations seem to be a promising concept to disentangle psychosocial determinants of fatigue in MS. Such knowledge would allow to improve the non-drug therapy and care of fatigued MS patients by development of adequate coping skills (in coping with stressful experience, timely diagnosis), of emotional distress and anxiety, also through psychotherapeutic and behavioral interventions, and the creation of social networks to support patients (54).

However, there are several limitations of currently available personality findings. First, we have no reliable premorbid data on personality structure in individuals suffering from MSF. Therefore, we do not know whether the personality profile as assessed after the onset of MS has not been influenced by depressive mood and/or other disease-related factors. Stressful situations as seen in MS may enhance or alter pre-existing personality traits and features and even lead to pathological states in terms of a personality disorder (54). Longitudinal study designs are needed to substantiate whether and possibly how personality structure is altered by MS disease. Second, data relating personality profile to fatigue are solely based on subjective ratings reported in fatigue questionnaires, i.e., assessed as subjective fatigue or fatigability over extended time (trait fatigue), but not on performance measurement after challenging mental and physical effort (state fatigue). This favors a bias towards psychological and trait aspects of fatigue. It remains to be established whether personality profiles show any correlation with test settings including objective measures of fatigue, i.e., reaction times, grip tests, and effort-related changes in performance.

When trying to draft a unifying hypothesis from the current findings, one could argue that there are two types of fatigue: (1) primary fatigue (intrinsically disease-related) and (2) a secondary form related to comorbid conditions. Fatigue in initial stages of MS might largely be driven by factors associated with disease coping while fatigue in later stages should predominantly be caused by inflammatory influence on the brain and functional consequences of brain lesions. Then, the two main subtypes of fatigue states, one “psychosocial” in origin and one characterized by “altered brain function” as formulated by S. Johnson (55) should be coexisting entities in the individual patient thus integrating multiple sources of origin. The main psychological factors interacting with fatigue that have been delineated so far are depression, anxiety, and inadequate disease coping. They seem to be related to personality profile and foster “maladaptive” reactions to MS diagnosis. On the other hand, disease status and disease progression are important physical factors. Therefore, disease-intrinsic determinants and extrinsic ones, that are not directly disease-related, might interact in the generation of fatigue in MS. Finally, psycho-biological models of fatigue (56), integrative physiological concepts like that of “central fatigue” (57) stressing the importance of abnormal patterns of activation in specific brain areas (58, 59) as well as the concept of enhanced cognitive reserve as a putative protective factor (60) are not exclusive, but complementary explanatory models of fatigue in MS. Their contribution to fatigue may change in every individual fatigued MS patient. Thus, the dichotomy of “physical” and “psychological” determinants of MS fatigue and the hitherto conflicting results may be reconciled by the view of fatigue representing a “multifaceted syndrome” with different mechanisms of origin (61).

## Conflict of Interest Statement

The authors declare that the research was conducted in the absence of any commercial or financial relationships that could be construed as a potential conflict of interest.
